# Akt1 Intramitochondrial Cycling Is a Crucial Step in the Redox Modulation of Cell Cycle Progression

**DOI:** 10.1371/journal.pone.0007523

**Published:** 2009-10-21

**Authors:** Valeria Gabriela Antico Arciuch, Soledad Galli, María Clara Franco, Philip Y. Lam, Enrique Cadenas, María Cecilia Carreras, Juan José Poderoso

**Affiliations:** 1 Laboratory of Oxygen Metabolism, University Hospital, University of Buenos Aires, Buenos Aires, Argentina; 2 Department of Organic Chemistry, Faculty of Exact and Natural Sciences, University of Buenos Aires, Buenos Aires, Argentina; 3 Laboratory of Motor Neuron Biology, Burke Medical Research Institute, University of Cornell, New York, New York, United States of America; 4 Department of Pharmacology and Pharmaceutical Sciences, School of Pharmacy, University of Southern California, Los Angeles, California, United States of America; 5 Department of Clinical Biochemistry, University Hospital, School of Pharmacy and Biochemistry, University of Buenos Aires, Buenos Aires, Argentina; University of Melbourne, Australia

## Abstract

Akt is a serine/threonine kinase involved in cell proliferation, apoptosis, and glucose metabolism. Akt is differentially activated by growth factors and oxidative stress by sequential phosphorylation of Ser^473^ by mTORC2 and Thr^308^ by PDK1. On these bases, we investigated the mechanistic connection of H_2_O_2_ yield, mitochondrial activation of Akt1 and cell cycle progression in NIH/3T3 cell line with confocal microscopy, *in vivo* imaging, and directed mutagenesis. We demonstrate that modulation by H_2_O_2_ entails the entrance of cytosolic P-Akt1 Ser^473^ to mitochondria, where it is further phosphorylated at Thr^308^ by constitutive PDK1. Phosphorylation of Thr^308^ in mitochondria determines Akt1 passage to nuclei and triggers genomic post-translational mechanisms for cell proliferation. At high H_2_O_2_, Akt1-PDK1 association is disrupted and P-Akt1 Ser^473^ accumulates in mitochondria in detriment to nuclear translocation; accordingly, Akt1 T308A is retained in mitochondria. Low Akt1 activity increases cytochrome *c* release to cytosol leading to apoptosis. As assessed by mass spectra, differential H_2_O_2_ effects on Akt1-PDK interaction depend on the selective oxidation of Cys^310^ to sulfenic or cysteic acids. These results indicate that Akt1 intramitochondrial-cycling is central for redox modulation of cell fate.

## Introduction

Akt (formerly protein kinase B) is a serine/threonine kinase implicated in the regulation of cell cycle progression, cell death, adhesion, migration, metabolism and tumorigenesis [1]. In 1991, collaborative studies resulted in the cloning of the viral oncogene *v*-akt. The predicted oncoprotein contained viral Gag sequences fused to a kinase related to protein kinase C [2]. Akt possesses an N-terminal pleckstrin homology (PH) domain (residues 1–113), a kinase domain (residues 150–408) that is similar to those found in other AGC members, such as cAMP-dependent protein kinase and protein kinase C, and a C-terminal regulatory domain (residues 409–480) containing a hydrophobic motif [2]. Akt defines a family of closely related, highly conserved cellular homologues [3]. In human, these are designated Akt1, Akt2, and Akt3, (PKB α, β and γ) and are located at chromosomes 14q32, 19q13 and 1q44, respectively [4].

Akt kinases are classically activated by engagement of receptor tyrosine kinases by peptide growth factors and cytokines, as well as oxidative stress and heat shock. Akt activation depends on PtdIns-3,4,5-P_3_ and to a lesser extent on PtdIns-4,5-P_2_, which are products of phosphoinositide 3-kinase [PI3K, 5]. The interaction of PtdIns-3,4,5-P_3_ with the PH domain of Akt1 favors the interaction with their upstream activators and its phosphorylation at two sites: one in the activation loop (Thr^308^) and another in the C-terminal tail (Ser^473^). Phosphorylation at Ser^473^ appears to precede and facilitate phosphorylation at Thr^308^ [6]. Akt1 is phosphorylated in Ser^473^ by subunits SIN1 and MIP1 of mTORC2 complex [rapamycin]-[insensitive companion of mTOR, 7, 8] while the kinase responsible for phosphorylation in Thr^308^ is PI3K-dependent kinase 1 [PDK1, 9]. Of note, the S473D mutant of Akt1 and Akt1 phosphorylated in Ser^473^ by the rictor-mTOR complex are better targets of PDK1 than nonphosphorylated Akt1 [6]. These findings suggest that phosphorylation at Ser^473^ may provide a docking site for PDK1 [10]. Once activated, Akt not only phosphorylates an ever-expanding list of substrates in cytosol but also can translocate to nuclei and mitochondria [11]. Furthermore, our group has previously demonstrated that mitochondrial Akt2 is an essential mediator for the regulation of muscle O_2_ utilization upon insulin stimulation [12].

Recent data strongly suggest that in addition to signaling cascades initiated by hormones or growth factors, reactive oxygen species (ROS) are involved in physiological signaling pathways that regulate a variety of cellular functions and hydrogen peroxide (H_2_O_2_) is the main messenger molecule [13], [14]. Mitochondria are suitable as a point of integration for these signaling pathways due to their critical role in cellular metabolism, redox balance, and survival-death mechanisms.

Research on mitochondria has focused on bioenergetics, biogenesis and the regulation of apoptotic cell death through mechanisms which have been conserved through evolution [15]–[17]. Proliferation is associated with low mitochondrial respiration [the Warburg effect, 17] and tumoral mitochondria only retain 10–50% of the activity of complexes I, II–III, and IV of quiescent tissues [18]. Akt can increase or decrease mitochondrial respiration [12], [19] but in spite of extensive research on death mediators and survival mechanisms [20], little is known about cell communication in terms of Akt trafficking into the organelles, the basis for intramitochondrial signaling.

In the present work, we adopted a novel approach to elucidate whether Akt1 sequential phosphorylation is related to the modulation of cell fate by its redox state. We demonstrate that the second phosphorylation of Akt1 in Thr^308^ occurs in mitochondria and that this effect is blunted at high redox state, thus eliciting different responses, either proliferation or apoptosis in the NIH/3T3 cells.

## Results

### The fate of NIH/3T3 cells depends on the redox status

**Figure 1:**
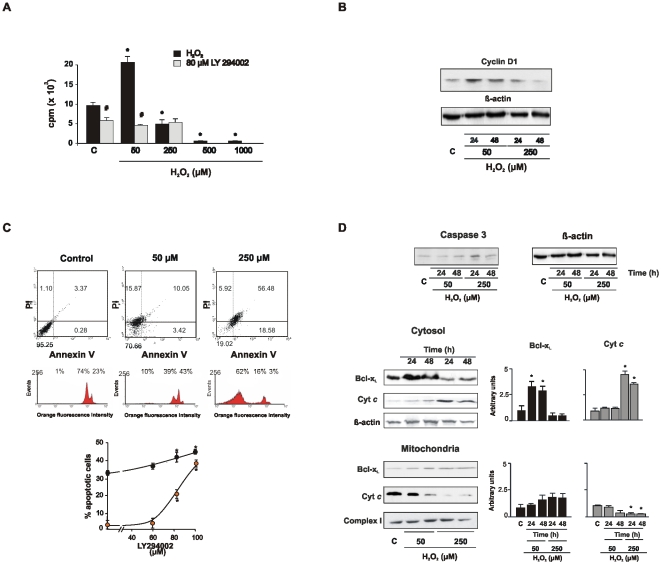
Hydrogen peroxide is the single signal that triggers cell proliferation or conducts to apoptosis. (A) H_2_O_2_ modulates cell proliferation rate through the Akt pathway; [^3^H] thymidine incorporation was measured 48 h after supplementing cells with H_2_O_2_ (C = control). Data are mean ± s.e.m; n = 8, experiment representative of 5, *p<0.05 *vs* C. When appropriate, cells were preincubated 2 h prior to redox stimulation with 80 µM PI3K/Akt inhibitor (LY294002) (^#^ *p*<0.05 *vs* H_2_O_2_ inhibition). The inhibitor reduced the proliferation rate by 50–75% between 0 and 50 µM H_2_O_2_, thus indicating dependence on Akt of redox effects on cell proliferation. (B) The redox variations of cyclin D1 paralleled those of the proliferation rate. (C) At higher concentration of H_2_O_2_, apoptosis increased by 10 fold as determined by flow cytometry with Annexin V staining (upper panel) and propidium iodide (medium panel) 48 h after H_2_O_2_ treatment. In the lower panel, the relationship between Akt inhibition and apoptosis is represented (red circles correspond to 50 µM H_2_O_2_ and black ones to 250 µM H_2_O_2_). (D) Expression of active caspase 3 (upper panel) and translocation of Bcl-x_L_ and cytochrome *c* from mitochondria (medium panel) to cytosol (lower panel) were determined 24 and 48 h after H_2_O_2_ , as markers of mitochondrial apoptotic pathways.

To test contextual effects of varying redox status, NIH/3T3 cells were incubated with 50–1000 µM H_2_O_2_ for 24–48 h. At 50 µM H_2_O_2_, cell proliferation rate doubled (Fig. 1A) and cyclin D1 expression was up-regulated (Fig. 1B); proliferation rise at low H_2_O_2_ was hindered by the PI3K inhibitor LY294002, thus indicating that cell duplication mainly occurred through the PI3K-Akt pathway (Fig. 1A). Conversely, 250 µM H_2_O_2_ caused a decrease of cell proliferation and cyclin D1 expression (Fig. 1A and B); proliferation was completely abrogated at 1 mM H_2_O_2_. Annexin V-propidium iodide double-positive cells and hypodiploid peak in flow cytometry indicated that high H_2_O_2_ concentration triggered apoptosis (Fig. 1C and S1, Methods S1). Irrespective of the H_2_O_2_ concentration, the percentage of apoptosis was increased by LY294002 inhibition of PI3K (Fig. 1C). Apoptosis was achieved upon activation of the mitochondrial caspase-3-dependent pathway resulting in the release of cytochrome *c* to cytosol (Fig. 1D) and concomitant loss of mitochondrial membrane potential (Fig. S2 and Table S1, Methods S2 and S3). Redox changes were accompanied by variations in the redistribution of Bcl proteins; there was retention of the antiapoptotic protein Bcl-x_L_ in mitochondria upon high redox stimulation (Fig. 1D). These results indicate that a) at low H_2_O_2_ proliferation is stimulated in NIH/3T3 cells *via* Akt and b) at high oxidant level Akt is down-regulated and the mitochondrial proapoptotic pathways are activated.

### The kinetics of cell trafficking and Akt1 redistribution upon redox stimuli

Akt is at the crossroad of several mitochondrion-mediated cell death pathways and constitutes an important target for cancer therapy. Here we show that cell cycle modulation by H_2_O_2_ is orchestrated by the Akt pathway in mitochondria. It has been previously reported that insulin-like growth factor 1 results in rapid translocation of P-Akt into mitochondria of neuroblastoma and human embryonic kidney cells [11].

**Figure 2:**
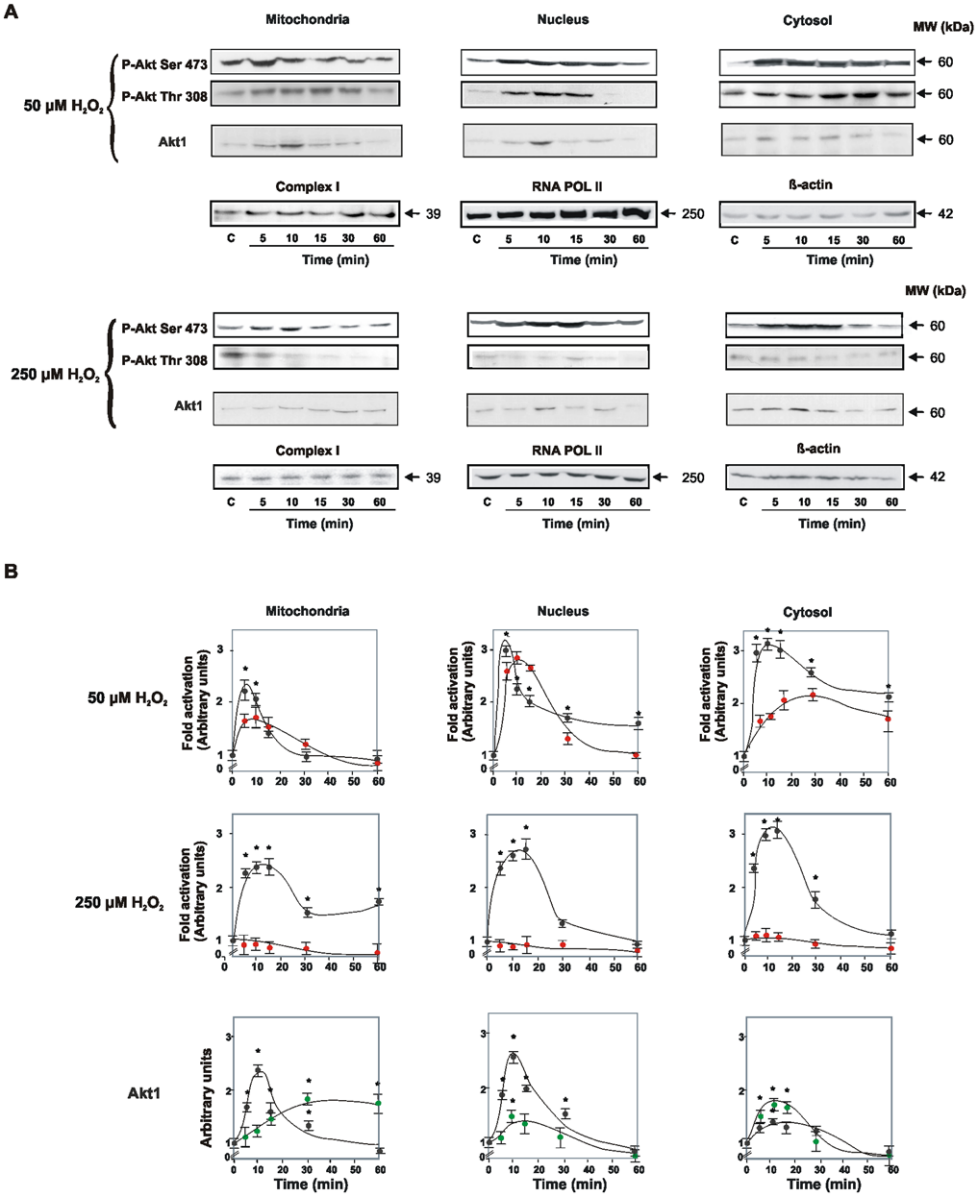
Kinetics of Akt1 activation and subcellular redistribution upon redox stimuli. In (A) and (B), western blots and curves represent the differential subcellular traffic, mitochondrial retention and activation of Akt1 at the proliferating and the apoptotic phases. Kinetics of the temporal distribution of P-Akt1Ser^473^ (black circles) and P-Akt1Thr^308^ (red circles) in the subcellular fractions are followed at 50 and 250 µM H_2_O_2_. Total Akt1 was evaluated in the same redox conditions (black circles correspond to 50 µM H_2_O_2_ and green ones to 250 µM H_2_O_2_). In (B), each point integrates densitometries from three separate experiments; *p<0.05. Protein loading was determined with antibodies anti complex I for mitochondria, β-actin for cytosol, and RNA POL RPB6 for nuclei.

The mitochondrial contribution in Akt1 activation was examined by redistribution of cellular Akt1 at the different H_2_O_2_ concentrations (Fig. 2A and B). Cell exposure to low H_2_O_2_ concentration caused a prompt appearance of Akt1 in mitochondria, cytosol, and nuclei that further decayed to the basal level. At high H_2_O_2_, Akt1 content increased slowly and largely retained in mitochondria, along with a discrete increase in cytosol and nuclei. To test whether a deficient traffic of Akt1 to mitochondria and nuclei depends on abnormal phosphorylations at Ser^473^ or Thr^308^, we compared the respective kinetics at the different redox status. At low H_2_O_2_, the kinetics of activation and redistribution of P-Akt1 Ser^473^ and P-Akt1 Thr^308^ mimicked those of total Akt1 (Fig. 2A and B); a slight delay of P-Akt1 Thr^308^ peak agreed with the conventional sequence for the two phosphorylation events [6]. Instead at high H_2_O_2_, phosphorylation of Thr^308^ was almost undetectable either in mitochondria or nuclei. Moreover, the peak of phosphorylation at Ser^473^ was not essentially modified though delayed and, P-Akt1 Ser^473^ was retained in mitochondria during the entire procedure (Fig. 2A and B). It is worthnoting that a) phosphorylation at Thr^308^ stimulates the rate of mitochondrial uptake of P-Akt1 Ser^473^; b) the retention of monophosphorylated P-Akt1 Ser^473^ in mitochondria and slow redistribution to nuclei reveal the interdependence of the two compartments; c) in this framework, experimental data clearly reveals the sequence of the subcellular traffic of Akt1: mitochondria to nucleus, and d) the second mitochondrial Akt1 phosphorylation in Thr^308^ drives the NIH redox transition from proliferation to apoptosis (Fig. 1C). No contamination of the different fractions was detected as assessed by western blot and enzymatic activities measurement (Fig. S3 and Table S2, Methods S4).

### Akt1 interacts with upstream PDK1 in mitochondria

**Figure 3:**
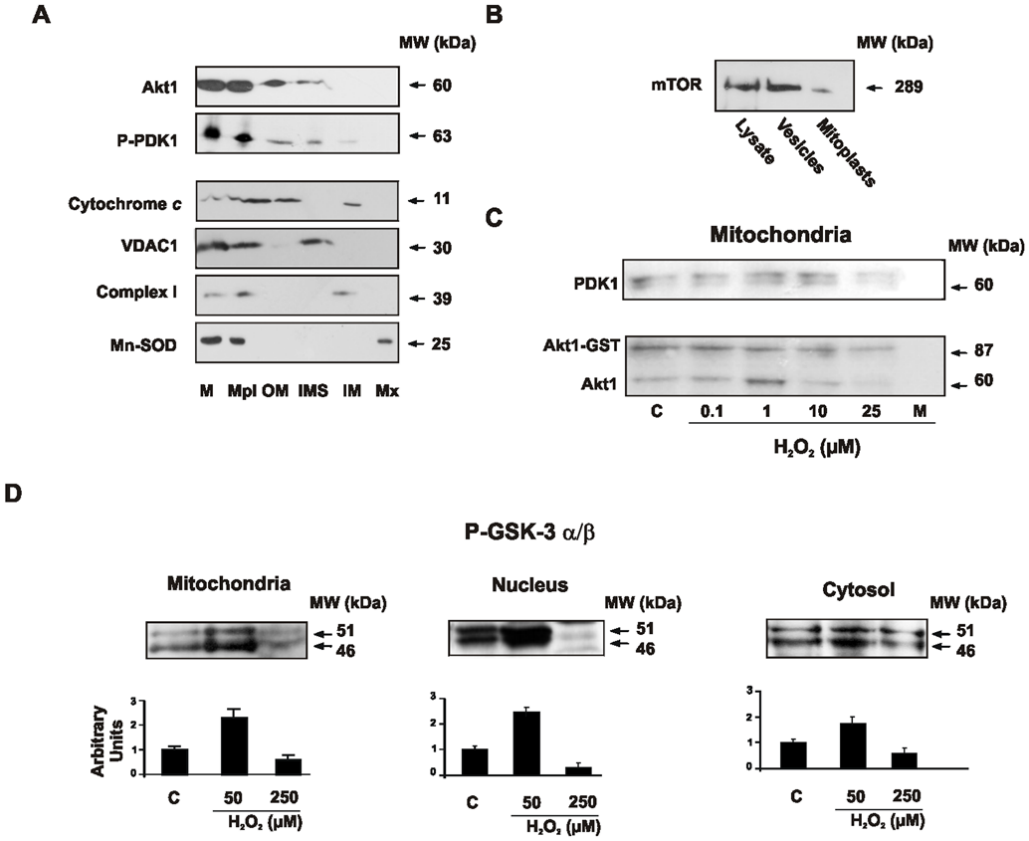
Akt1 activity relies on the H_2_O_2_-dependent adjustment of the binding to P-PDK1 in mitochondria. (A) Akt1 and P-PDK1 are localized in the mitochondrial outer membrane and the intermembrane space. Submitochondrial localization was assessed by western blot. (M: mitochondria; Mpl: mitoplast; OMM: outer mitochondrial membrane; IMS: intermembrane space; IMM: inner mitochondrial membrane; Mx: mitochondrial matrix). Identity of mitochondrial fractions was corroborated with specific antibodies anti complex I 39 kDa subunit, voltage-dependent anion channel (VDAC1), superoxide dismutase II and cytochrome *c*. (B) mTORC2 is mainly localized in vesicle fractions. (C) P-PDK1 and Akt1 interaction is enhanced at low H_2_O_2_ condition while binding is abolished at high redox status. Human recombinant Akt1-GST was immobilized on agarose, oxidized with H_2_O_2_ and incubated with mitochondrial fraction. P-PDK1 was detected by western blot. (D) Akt1 activity is modulated by the redox status. The activity was detected through the level of phosphorylation of GSK-3 α/β substrate in mitochondria, cytosol and nuclei.

It was recently established that Akt1 phosphorylation at Ser^473^ is catalyzed by mTORC2 complex [8], [20] while phosphorylation at Thr^308^ is catalyzed by P-PDK1 [9]. Both phosphorylations are thought to occur in the cell plasma membrane as driven by Pleckstrin homology domains that interact with PI3K in the hydrophobic phase. However, after mitochondrial sub-fractionation we found both Akt1 and upstream P-PDK1 in the mitochondrial outer membrane and intermembrane space of NIH/3T3 cells; PDK1 was also present in the inner mitochondrial membrane (Fig. 3A). Instead, mTORC2 was expressed in plasma membrane and cytosol but poorly expressed in mitochondria (Fig. 3B). To explore the effects of H_2_O_2_ on Akt1-PDK1 interaction, we performed pull-down assays with human recombinant Akt1-GST bound to agarose beads, previously treated with 0.1–25 µM H_2_O_2_, and subsequently incubated with the mitochondrial fractions. Akt1-GST binding to PDK1 was enhanced at low H_2_O_2_ level (1 µM H_2_O_2_) while oxidation of Akt1-GST exposed to high H_2_O_2_ yield (10 µM H_2_O_2_) disrupted the Akt1-PDK1 interaction (Fig. 3C). To evaluate the redox effects on Akt1 activity in the same conditions, we immunoprecipitated Akt1 from cytosolic, mitochondrial and nuclear extracts of NIH cells treated with different H_2_O_2_ concentrations and measured the formation of P-GSK-3 α/β in an *in vitro* assay. In agreement with the former result on Akt interaction with PDK1, Akt1 activity in the organelles was enhanced by 150% at low H_2_O_2_ and decreased to 45% of control at high H_2_O_2_ yield (Fig. 3D).

### Akt1 traffic to the nucleus requires phosphorylation at Thr^308^ in mitochondria

**Figure 4:**
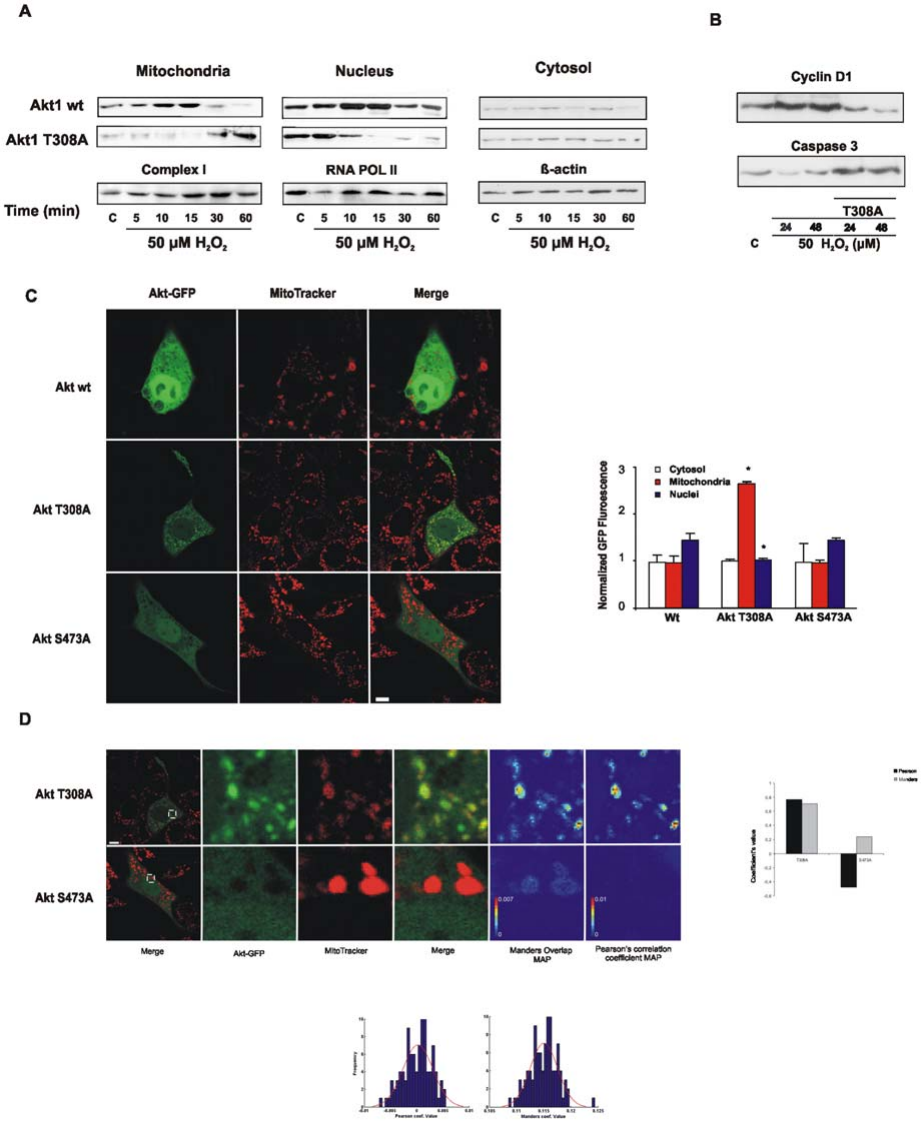
Complete activation of Akt1 and efficient shuttle to nuclei critically depend on mitochondrial phosphorylation of Thr^308^. (A) Akt mutation in Thr^308^ causes kinase retention in mitochondria and prevents final shuttle to nucleus. Kinetics of differential mitochondrial, cytosolic and nuclear distribution of transfected wild type and T308A Akt were followed by western blot after 50 µM H_2_O_2_ treatment. (B) Prevention of Akt1T308A translocation to nucleus causes a decrease in cyclin D1 expression and a rise in caspase 3 level. (C) NIH/3T3 cells were transfected with Akt1-GFP, Akt1 S473A-GFP or Akt1 T308A-GFP and stained with MitoTracker Deep Red. Images of GFP and MitoTracker fluorescence intensity individual and merged channels are shown. Bar  = 10 µm. GFP mean fluorescence intensity was quantified in cytosol, mitochondria or nuclei and normalized to whole cell mean GFP fluorescence. In (D), zoom of the green, red and merged images. Manders overlap and Pearson's correlation coefficient maps are included. Color bar  =  single pixel contribution to the overall coefficient. On the right panel, statistical analysis of hAkt1 localization to mitochondria. Upper panels: the probability distribution of random colocalization was obtained by computing the the Pearson's correlation coefficient (21) or Manders overlap coefficient (Villalta *et al*., in preparation) after repetitively scrambling the pixel positions in the green hAkt1T308A-GFP zoomed image. Red line  =  normal distribution adjusted to the data. Lower panel: Manders overlap and Pearson's correlation coefficient values were estimated for the zoomed images.

To assess Akt1 mitochondria-nucleus functional connection and the significance of intramitochondrial Akt1 phosphorylation by PDK1, we obtained Akt1 T308A by directed mutagenesis, which renders a non-phosphorylatable mutant at Thr^308^. Cells transfected with wt Akt1 and stimulated with 50 µM H_2_O_2_ behaved similarly to the previous results (Fig. 4A); Akt1 translocation was maximal at 5–10 min and further decreased in mitochondria while increased in the nuclei. On the contrary, Akt1 T308A accumulated in mitochondria and did not translocate to but rather decreased in nuclei suggesting that complete activation of Akt1 and further shuttle to nuclei depend on the phosphorylation in Thr^308^ by mitochondrial PDK1 (Fig. 4A). Accordingly, Akt1 T308A transfected cells did not elicite an increase in cyclin D1 expression whereas the apoptotic machinery was activated *via* caspase-3 (Fig. 4B).

Real time video imaging confirmed this Akt1 distribution at the different times (Fig. 4C, S2 and Videos S1–S4). In these experiments, NIH/3T3 cells were transfected with wt Akt1-GFP or the respective mutants that lack one of the phosphorylation sites, Akt1 T308A-GFP and Akt1 S473A-GFP, and further stained with a specific mitochondrial marker, MitoTracker Deep Red, and analyzed by confocal microscopy. In these conditions, stimulation with 50 µM H_2_O_2_ differently redistributed wt Akt1-GFP and the mutants among the different subcellular compartments (Fig. 4C, S2 and Videos S1-S4). Wild type Akt1-GFP traversed mitochondria rapidly and localized predominantly in nuclei (intense green nuclei) whereas Akt1 S473A-GFP localization to nuclei and mitochondria was modest. Akt1 T308A-GFP was accumulated in mitochondria during 0–15 min in detriment to its nuclear localization. Akt1 T308A-GFP preferential retention in mitochondria and the scarce Akt1 S473A-GFP presence in the organelle were appreciated in enlarged views of the images (Fig. 4D). In addition, the Pearson's correlation coefficient and Manders overlap coefficient maps clearly show a higher contribution to the colocalization of the pixels enclosed in the mitochondrial region (intense red color), which indicates that those pixels display high green (GFP) and red (MitoTracker) fluorescence intensity (details in Methods S5). The coefficient value was further determined for the images in figure 4D. For Akt1 T308A-GFP both coefficient values were positive and high and significantly above those expected for random generated images (see distribution coefficients for random images in figure 4D, bar graphs) [21] which argues for true non-fortuitous superposition of randomly distributed fluorophores. Instead, the coefficients resulted near zero (Manders overlap) or negative (Pearson's correlation) for Akt1 S473A-GFP, which indicate lack or minimal colocalization. These results confirm that the sequential phosphorylation of Akt1 is strictly necessary for the regulation of the kinase redistribution among the cellular compartments: Ser^473^ phosphorylation is central for kinase translocation to mitochondria and mitochondrial Thr^308^ phosphorylation is crucial for Akt1 traffic to nucleus.

### Akt1 requires to be phosphorylated at Ser^473^ to enter mitochondria “*ex vivo*”

**Figure 5:**
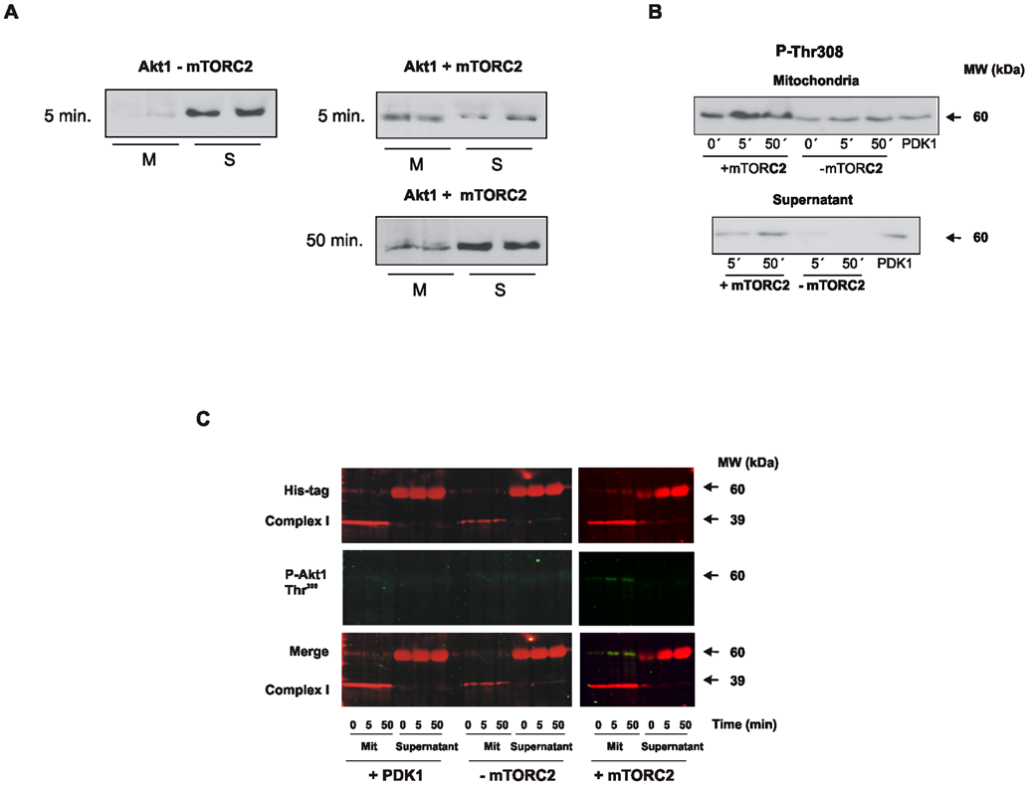
The mitochondrial cycle of Akt1 under redox stimuli. In (A) and (B), Akt1 requires to be phosphorylated in Ser^473^ to enter mitochondria. Inactive Akt1-His tagged and mTORC2 were incubated in kinase buffer to allow phosphorylation. In absence of P-mTORC2 Akt1 remains outside mitochondria, In the presence of mTORC2, P-Akt Ser^473^ translocates to mitochondria and becomes phosphorylated in Thr^308^; approximately after 50 min biphosphorylated P-Akt Ser^473^/Thr^308^ becomes detectable in the supernatant. These extracts were incubated with purified mitochondria in import buffer and the samples were centrifuged and prepared to run in SDS-PAGE. (C) Imaging of wb using a His-tag ab anti mouse conjugated to Cy3 and a P-Thr308 ab anti rabbit conjugated to Cy2 shows colocalization (yellow) in the presence of P-mTORC2. Addition of PDK1 alone is unable to phosphorylate Akt1 in Thr^308^.

To elucidate the complete cycle of Akt1 phosphorylation, recombinant hAkt1-his tagged was incubated with isolated NIH mitochondria before and after being monophosphorylated *in vitro* with brain mTORC2. It is shown here (Fig. 5A and B) that inactive Akt1 cannot enter to isolated energized mitochondria. Instead, P-Akt1 Ser^473^ enters mitochondria very fast up to a relative rate of 1.2 pg/min.μg prot. An almost complete decay of mitochondrial P-Akt1 Ser^473^ was observed in the isolated organelles at 50 min. By that time, Akt1 concentration increased in the supernatant by one-fold and the data indicated a reverse in the net flux of Akt1 among the two compartments. Furthermore, we examined whether mtP-Akt1 Ser^473^ had been modified in isolated mitochondria, *i.e.*, by acquiring the second phosphorylation. By utilizing the antibody anti P-Thr^308^, we confirmed that P-Akt1 Ser^473^ had been phosphorylated in mitochondria to P-Akt1 Ser^473^/Thr^308^, the completely active variant of the kinase (Fig. 5A and B).

### Mitochondria and plasma membrane cooperate for complete Akt1 activation *in vitro* in reconstituted vesicles

**Figure 6:**
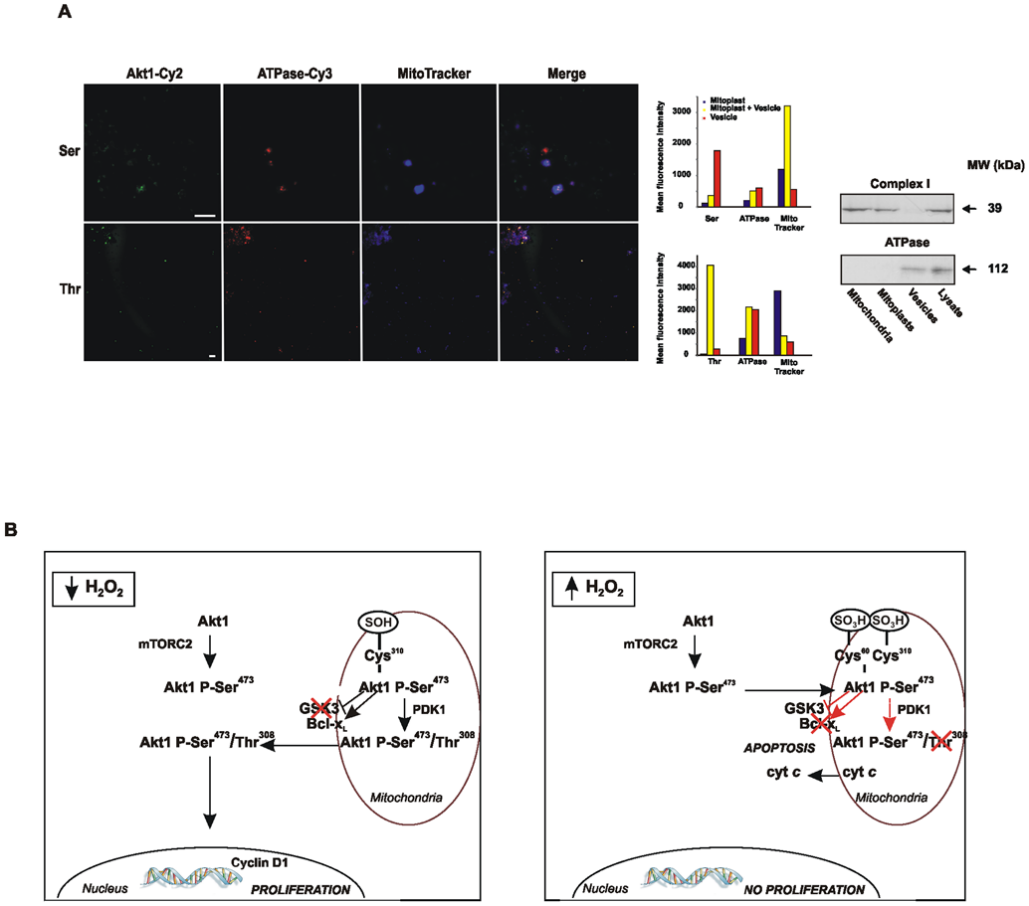
The sinergy between plasma and mitochondrial membranes in Akt double phosphorylation. (A) Second Akt1 phosphorylation in Thr^308^ occurs in mitoplasts. Vesicles and mitoplasts were isolated from NIH/3T3 cells, and co-incubated in the presence of Akt1 in an import assay (see methods). The preparations were labeled with MitoTracker Deep Red to identify mitoplasts and further fixed and labeled with an antibody against ATPase and a second antibody conjugated with Cy3 to recognize vesicles. P-Ser^473^ or P-Thr^308^ were labeled with specific antibodies and a second antibody conjugated with Cy2. Green (Akt), red (vesicles), blue (mitoplasts) and merged images are shown. Bar  = 10 µm. On the right, Cy2 (Ser or Thr), Cy3 (ATPase) and MitoTracker Deep Red fluorescence intensity was measured on every vesicle, mitoplast or fusion particle (vesicle + mitoplast) in the images (see methods). Particle populations were partitioned in accord to its prevalent label: mainly vesicles (red bar), mainly mitoplasts (blue bar) or both stains (yellow bar) using k-means clustering method. Cy2 fluorescence intensity was compared in these three populations. (B) Scheme of Akt1 sequential phosphorylation, activation and consequent subcelullar redistribution under opposite redox states.

To confirm that mitochondria and plasma membrane are essential for Akt1 complete activation, inactive recombinant hAkt1 was subjected to oxidation by H_2_O_2_ (0.1 µM) and incubated with membrane vesicles purified from the NIH cells alone and with vesicles that engulfed a preparation of NIH mitoplasts in a proportion of 10–100 mitoplasts per vesicle. Vesicles were specifically labeled with an antibody that recognizes membrane ATPase and a secondary antibody conjugated with Cy3. Mitoplasts were specifically stained with MitoTracker Deep Red. The particles were also incubated with antibodies against P-Akt1 Ser^473^ or P-Akt1 Thr^308^ and secondary antibodies conjugated to Cy2. In the confocal microscope images, red particles account for vesicles, blue particles for mitoplasts and fusion particles are shown in magenta (Fig. 6A). P-Akt1 Ser^473^-Cy2 label was observed predominantly in vesicles that contained no or little mitoplast stain (Fig. 6A, bars) but contained mTORC2 (Fig. 3B). Instead, these vesicles did not cause P-Akt1 Thr^308^-Cy2. This fact indicates that a) the first Akt1 phosphorylation depends on plasma membrane; b) in the absence of mitochondria that participates in the phosphorilation of P-Akt1 Ser^473^-Cy2 at Thr^308^, the kinase is accumulated in the vesicles. Instead, we observed the highest P-Akt1 Thr^308^-Cy2 and the lowest P-Akt1 Ser^473^-Cy2 fluorescence intensity in those vesicles that contained predominant mitoplast stain (Fig. 6A, bars). Because the first phosphorylation does not occur in the absence of vesicles, mitoplasts by themselves were not capable to accomplish Thr^308^ phosphorylation. These results show that plasma and mitochondrial membranes cooperate for complete Akt activation in NIH cells, and confirm that Ser^473^ phosphorylation is a prerequisite for Thr^308^ phosphorylation to occur in mitoplasts. A scheme of double Akt1 phosphorylation in the different subcellular localizations is summarized in Figure 6B.

### Akt1 Cys^60^ and Cys^310^ are main targets for H_2_O_2_ oxidation

Mass spectrometry analysis of recombinant Akt1 incubated *in vitro* with different H_2_O_2_ concentrations (representative of those utilized in the *in vivo* assays) are shown in Table 1. In non-stimulated cells only Cys^310^ (which is adjacent (∼5 Å) to Thr^308^) was oxidized to sulfenic acid. With increasing H_2_O_2_ concentrations, Cys^310^ remained partly as sulfenic acid (Cys-SOH) and partly oxidized further to cysteic or sulfonic acid (Cys-SO_3_^3−^). At 1 µM H_2_O_2_ Cys^310^ was always modified as cysteic acid. In addition, Cys^60^ in the PH domain was not modified in non-stimulated cells or at very low H_2_O_2_ but resulted every time oxidized to cysteic acid from 0.1 to 20 µM.

**Table 1:** Mass spect of oxidized recombinant Akt1.

H_2_O_2_ (μM)	tryptic peptide	residue	prob.	MSc*	modification	Δcn**	peptide charge
none	EAPLNNFSVAQCQLMK	Cys^60^	95%	75.5	–	0.55	2
	TFC^+16^GTPEYLAPEVLEDNDYGR	Cys^310^	71%	34.2	Sulfenic acid (+16)	−0.40	2
0.001	EAPLNNFSVAQCQLMK	Cys^60^	95%	75.5	–	0.55	2
	EAPLNNFSVAQCQLMK	Cys^60^	95%	66.6	–	0.038	2
	EAPLNNFSVAQCQLMK	Cys^60^	95%	66.3	–	0.33	2
0.01	EAPLNNFSVAQCQLMK	Cys^60^	95%	75.51	–	−0.79	2
	TFC+^16^GTPEYLAPEVLEDNDYGR	Cys^310^	94%	41.25	Sulfenic acid (+16)	−0.58	2
	TFC^+48^GTPEYLAPEVLEDNDYGR	Cys^310^	95%	57.2	Sulfenic acid (+16)	0.43	2
	TFC^+48^GTPEYLAPEVLEDNDYGR	Cys^310^	88%	39.06	Cysteic acid (+48)	0.066	2
0.1	EAPLNNFSVAQCQLMK	Cys^60^	95%	77.4	–	−0.17	2
	EAPLNNFSVAQC^+48^QLMK	Cys^310^	95%	65.8	Cysteic acid (+48)	0.79	2
	TFC^+48^GTPEYLAPEVLEDNDYGR	Cys^310^	88%	45.4	Cysteic acid (+48)	−0.33	2
1	TFC^+48^GTPEYLAPEVLEDNDYGR	Cys^310^	95%	62.9	Cysteic acid (+48)	0.76	2

hAkt1 (1 µg/50 µl) was oxidized by 15 min in 1X kinase buffer (Calbiochem Cat# CBA055); *MSc: Mascot Ion Index; **Δcn stands for the difference in the cross-correlation score between the top two candidates peptides for a given input data file.

## Discussion

The redox modulation of NIH/3T3 cell fate entails translocation of Akt1 to mitochondria. This fact defines Akt1 cellular dynamics in a three compartmental signaling pathway: mitochondria↔cytosol↔nucleus, with further effects on the progression of cell cycle or on apoptosis. A redox connection between Akt1 and mitochondria has been formerly revealed by Nogueira *et al*. [18] in MEFs cells where wt myrAkt transfection increased the respiratory rate and promoted high 2′,7′-dichlorofluorescein-diacetate (DCFH-DA) fluorescence due to high oxidant yield; in this condition, Akt inhibition with antitumoral rapamycin-PEITC association revealed cell switching to apoptosis. It is surmised that irrespective of concentration, Akt uses and drives H_2_O_2_ as a single mitochondrial signal to control its final effects on the cell cycle.

The bases for Akt activation at low H_2_O_2_ relied on the efficient sequential biphosphorylation at Ser^473^ and Thr^308^. Low redox stimuli thereby acts by increasing the mitochondrial availability of monophosphorylated P-Akt1 Ser^473^ which depends on the extramitochondrial activation of mTORC2 by PI3K [8]. mTORC2 expression is rather poor in mitochondria and therefore Akt1 Ser^473^ is mostly phosphorylated outside the organelles. Recently, Alessi *et al*. reported an additional phosphorylation by mTORC2 in Akt Thr^450^ in the turn motif that protects the hydrophobic motif from dephosphorylation and increases the stability of P-Akt1 Ser^473^ [22]. mTORC2 activity is therefore associated here to fast P-Akt1 Ser^473^ entrance to mitochondria with prompt exit of Akt1 Ser^473^/Thr^308^ towards the nucleus. Moreover, unphosphorylated Akt1 cannot enter to isolated mitochondria (Fig. 5) and, on the contrary; solely P-Akt Ser^473^ is easily translocated to the organelles *ex vivo* being found after a few minutes in the supernatant as P-Akt1 Ser^473^/Thr^308^ (Fig. 5). In normal cells, mitochondrial availability of P-Akt Ser^473^ is modulated as well by the activity of antitumoral PI3K phosphatase PTEN. It is noticed that PTEN is reversibly inactivated in the NIH cells by 50–100 µM H_2_O_2_, through the oxidation of Cys^124^ [23].

Under hormone stimulation, a similar kinetics was found by Bijur and Jope who reported rapid insulin-induced translocation of P-Akt Ser^473^ to energized mitochondria from SKYH cells; the authors identified several target proteins that underwent Akt-dependent phosphorylation, including GSK-3 α/β and a subunit of ATP synthase [11]. Our group reported a similar translocation of Akt1 and Akt2 to muscle mitochondria by long-lasting insulin that ended in the phosphorylation of mitochondrial nitric oxide synthase (mtNOS) and GSK-3 α/β, with significant changes in glucose metabolism [12].

Conversely, high H_2_O_2_ concentration resulted in disruption of the second phosphorylation of Akt at Thr^308^ in mitochondria and in the consequent accumulation of the monophosphorylated P-Akt1 Ser^473^ with very low activity in the organelles. The apoptotic response to high H_2_O_2_ is complex and involves both the activation of proapoptotic pathways (release of cyt. *c*, Fig. 1) and loss of repression of apoptosis (the survival signal) due to Akt1 (activation or inhibition of antiapoptic Bcl-x_L_ at the different redox states; Fig. 1). It may be surmised that the multiplicity of redox mechanisms that trigger mitochondrial apoptosis is centered on the weak inhibitory effects of monophosphorylated Akt in the organelles; at the same H_2_O_2_ concentration, the higher percentage of apoptotic cells was achieved at maximal inhibition of PI3K/Akt pathway (Fig. 1). Therefore, a marked increase of GSK-3 α/β activity due to weak Akt1 activation (Fig. 3) contributed to NIH/3T3 cell apoptosis (Fig. 1); proapoptotic GSK3 α/β activity is abolished by phosphorylation at Ser^23^ and Ser^9^, mainly catalyzed by Akt in mitochondria [24]. Otherwise, prevention of staurosporin-induced apoptosis by Akt1 is a protective mechanism that involves the activation of Bcl-x_L_ and reciprocally, this protein promotes or restores the Akt1 activity [25]. The antitumoral effects of rapamycin are due to the disruption of Akt and the decline of Bcl-x_L_. Inhibition of mTOR-p70S6 pathway and Akt1 by rapamycin dramatically inhibits transformation of NIH/3T3 cells in over-expressing constitutively active myr-Akt1 (3T3-Akt1 cells) or myr-Akt2 [3T3–Akt2 cells, 26].

The second phosphorylation of Thr^308^ is required for sustaining a significant traffic of mitochondrial Akt to nucleus. Mutant Akt T308A is rather limited in its entrance to nucleus and the precursor P-Akt1 Ser^473^ accumulates in mitochondria, thus mimicking the effects of high H_2_O_2_ concentration. Phosphorylation at Thr^308^ is PDK1-dependent [27] and completes the initial effect of mTORC2. The sequential phosphorylation of Ser^473^ and Thr^308^ has been previously reported *in vivo* [6]; disruption of PDK1 reduces phosphorylation of Thr^308^ *in vivo*, and PDK1 deficient mice are considerably smaller than wt animals [28]. We demonstrate here that PDK1 binds P-Akt1 Ser^473^ at the mitochondrial outer membrane and the intermembrane space and that PDK1 is constitutively active (P-PDK1) at this localization. Previous studies in NIH cells propose that PDK1 is permanently complexed to Akt at the PH domain in an inactive conformational state that might be turned into an active state by growth factors or, hypothetically, by redox status [29]. In addition and guided by the Peckstrin homology domains, Akt1 and PDK migrate to plasma membrane where PDK1 activity is markedly increased by PtdIns; Filippa *et al*. showed that it is P-PDK-1 that recruits Akt1 to the plasma membrane [30]. Interestingly, Connor et al. demonstrated that the mitochondrial membrane contains PtdIns as well and that alterations in the steady-state production of mitochondrial H_2_O_2_ modulate the redox state of PTEN [31]. The alternative amount of Akt1 or Akt-PDK1 migrating to mitochondrial or plasma membranes should depend on the stimuli (H_2_O_2_ produced in mitochondria or growth factors acting on plasma membrane TK receptors) and, under certain circumstances, Akt and PDK1 complex could be partitioned into the two membranes for cooperative effects. However in this work, membrane preparations obtained from NIH/3T3 cells and devoided of mitochondria contained P-PDK-1 but were unable to efficiently phosphorylate Akt1 Thr^308^ (Fig. 6).

The modulation of Akt1 binding to PDK1 depended on specific thiol oxidations (Table 1). In non-stimulated cells Akt1 Cys^310^ adjacent to Thr^308^ in the catalytic loop was basally oxidized to sulfenic acid (-SO^−^), a modification also observed at very low H_2_O_2_ concentration. In this context, we hypothesized that a disulfide bridge may stabilize Akt1-PDK1 binding thus favoring Thr^308^ phosphorylation at the very low H_2_O_2_ concentration. Otherwise, as it occurs in peroxiredoxins, an ATP reaction with Cys-SO^-^ may form a phosphoryl thiol that may finally transfer the phosphate to Thr^308^ [32]. Instead, moderate to high H_2_O_2_ concentration led to strong oxidation of Akt1 Cys^60^ in the PH domain, and Akt1 Cys^310^ to sulfonic acid (-SO_3_^2−^). In this case, negative charges likewise disrupt the PDK1 approach to the Akt1 Pleckstrin homology domain, and the advance of negatively charged ATP^3−^ to Thr^308^. In accord, C310A mutation in Akt inhibits its catalytic activity, an effect also obtained by supplementation with lactoquinomycin which acts on the Cys^310^–S^−^ group [33] Comparatively, ERK2 is as well oxidized at Cys^38^ and Cys^214^ to get an efficient binding with its upstream kinases MEK1/2 in mitochondria [34].

Mitochondrial contribution to proliferation and apoptosis has been revealed in the last decade. As shown here, redox modulation of cell fate involved Akt intramitochondrial signaling. This contribution is understood on the bases that a) mitochondria are the most important oxygen users and producers of oxidants like H_2_O_2_; b) double phosphorylation of kinases may represent a cooperative control of activation in redox modulation of metabolism.

It may be surmised that, Akt phosphorylation in mitochondria is not only a single step in kinase activation, but a modality in which cells select predominantly mitochondrial apoptotic or nuclear proliferative pathways (Fig. 6B). We reported that mitochondrial dysfunction is associated to low H_2_O_2_ yield and persistent proliferation in embryonic and transformed cells [34]. These effects imply that the disruption of intramitochondrial signaling in the activation of kinases might conduct to persistent proliferation and cancer or to premature cell death.

## Materials and Methods

### Cell line, culture conditions and treatments

NIH/3T3 cell line was maintained in Dulbecco's modified Eagle's medium (D-MEM, Gibco) supplemented with 10% bovine calf serum (BCS) and 50 µg/ml gentamycin at 37°C in a humidified 5% CO_2_ atmosphere. For treatment, cells were 24 h serum starved, and then stimulated with H_2_O_2_ and/or the Akt inhibitor (LY294002, Sigma) at the appropriate concentrations.

### Preparation of nuclear, mitochondrial and cytosolic fractions

NIH/3T3 cells were lysed in MSHE buffer (0.22 M mannitol, 0.07 M sucrose, 0.5 mM EGTA, 2 mM HEPES/KOH, 1 mM phenylmethylsulfonylfluoride (PMSF), 5 µg/ml leupeptin, 5 µg/ml pepstatin, 5 µg/ml aprotinin, 25 mM NaF, 1 mM sodium orthovanadate, pH 7.4. The homogenate was centrifuged for 10 min at 1000×*g* (pellet =  crude nuclear extract). The supernatant was centrifuged at 10000×*g* for 20 min; supernatant fraction (cytosol) was then collected and mitochondrial pellet fraction was resuspended in MSHE. The crude nuclear pellet was washed once with buffer A (10 mM Tris, 1.5 mM EDTA, 10% glycerol, 1 mM PMSF, 5 µg/ml leupeptin, 5 µg/ml pepstatin, 5 µg/ml aprotinin, 5 mM NaF, 1 mM sodium orthovanadate, pH 7.4) containing NP-40 0.01%. The washed crude nuclear pellet was then resuspended in buffer A plus 0.4 M KCl, and incubated for 30 min at 4°C. The suspension was centrifuged at 15000×*g* (30 min) and diluted with the same volume of buffer A to reduce salt concentration [33]. The purity of the fractions was assessed by Western blot with antibodies against complex I (mitochondria), β-actin (cytosol) and RNA POL II (subunit 250 kDa) (nuclei). Protein concentration was determined by Bradford method [35].

### Submitochondrial fractioning

Purified mitochondria were osmotically broken by diluting the mitochondrial pellet in four volumes of distilled water and centrifuged for 10 min at 12000×*g* to give a supernatant containing the mitochondrial outer membrane and the intermembrane space, and a mitoplast pellet (inner membrane enclosing the matrix). Then, the mitoplast fraction was sonicated twice at 40 W for 10 sec with a Cole-Parmer sonicator (WPI, Sarasota, FL, USA). Subsequently, samples were centrifuged for 10 min at 8000×*g* to precipitate unbroken mitochondria. This supernatant, together with the first step one, were centrifuged for 30 min at 100000×*g* obtaining inner membrane and matrix, and outer membrane with intermembrane space, in the pellet and supernatant of both fractions respectively [36].

### Proliferation assay

NIH/3T3 cells were seeded in a multiwell plate, serum starved for 24 h, and treated with H_2_O_2_ for another 48 h in the presence of 0.8 µCi/well of [^3^H] thymidine (specific activity, 70 to 90 Ci/mmol; NEN/Dupont, Boston, Mass.). Cells were then trypsinized and harvested. Assays were performed in octuplicate. Radioactivity was measured in a liquid scintillation counter (Wallac 1414, Turku, Finland, 33).

### Apoptosis assays

NIH/3T3 cells treated with H_2_O_2_, were harvested and incubated with (i) 100 mg/ml propidium iodide in 0.1% sodium citrate, 0.1% Triton X-100 at 4°C overnight in the darkness [37] or (ii) Annexin V-FITC (Immunotech) according to manufacturer's instructions. Cells were run on a FACScalibur flow cytometer (Becton-Dickinson, Mountain View, CA) and analyzed with WinMDI software for Windows.

### Western blot

Mitochondrial, nuclear (50 µg/lane each) and cytosolic proteins (25 µg/lane) were separated by electrophoresis on SDS-polyacrylamide gels and transferred to a PVDF membrane (GE Healthcare). Membranes were incubated with antibodies anti Akt1, P-Akt1 Ser^473^, P-Akt1 Thr^308^ (Cell Signaling), cytochrome *c*, complex I, His (Molecular Probes), Bcl-x_L_, cyclin D1, β-actin, RNA POL RPB6 (Santa Cruz) or caspase 3 and HA (Sigma). Secondary antibodies were conjugated to horseradish peroxidase (GE Healthcare). Detection of immunoreactive proteins was accomplished by chemiluminescence with ECL (GE Healthcare). Quantification of bands was performed by digital image analysis using a Hewlett-Packard Scanner and Totallab analyzer software (Nonlinear Dynamics Ltd, Biodynamics, Argentina). For Li-Cor detection system, membranes were incubated with goat anti-rabbit IRDye 800CW and goat anti-mouse IRDye 680 (Li-Cor Biosciences) and fluorescence was detected using the Oddysey Infrared Imaging System (Li-Cor Biosciences).

### Akt activity assay

Mitochondria were lysed in 50 mM Tris HCl pH 7.4, 0.1 mM EDTA, 0.1 mM EGTA and immunoprecipitation was carried out using an immobilized Akt1 antibody. After centrifugation, the pellet was washed twice in PBS and resuspended in kinase buffer (50 mM Hepes K^+^ pH 7.5, 150 mM NaCl, 1 mM EDTA, 2.5 mM EGTA, 50 mM NaF, 0.183 mg/ml sodium orthovanadate, 1 mM DTT and 0.1% Tween 20) supplemented with 1 µl of 10 mM ATP and 1 µg of GSK-3 fusion protein. The mix was incubated for 30 min. at 30°C and the reaction was finished with 25 µl 3x sample buffer. Finally, samples were run on SDS-PAGE and transferred to PVDF membranes. Membranes were incubated with a P-GSK-3 α/β antibody (Cell Signaling) and revealed as previously described.

### Pull down assay

Mitochondrial fractions were incubated in the presence of human recombinant Akt1-GST agarose (Cell Signaling) in lysing buffer for 2 h at 4°C. When appropriate, recombinant kinase was oxidized with H_2_O_2_. After incubation, agarose beads were precipitated, washed in lysing buffer and cracked in Laemmli-loading buffer. Finally, samples were analyzed by western blot as described above incubated with an antibody anti PDK1 (Cell Signaling) and revealed as previously mentioned.

### Transient transfections

Cells were seeded onto a 22.1 -mm diameter well and transiently transfected with 1 µg of pcDNA3 wild type Akt1, Akt1 S473A or Akt1 T308A (obtained by *in vitro* site-directed mutagenesis system, Promega). Transfections were carried out for 24 h in DMEM with 10% BCS without antibiotics utilizing Lipofectamine Reagent in Opti-MEM (Invitrogen). After transfection, cells were stimulated with 50 µM H_2_O_2_ for the indicated times before cells were harvested and prepared for subcellular fractionation. Samples were analyzed by western blot as above with an anti-HA antibody (Sigma).

### Phosphorylation and translocation assay

0.3 µg of recombinant inactive Akt1 (Prospec) and 0.8 µg of mTOR (Calbiochem) were incubated in kinase buffer (Calbiochem), supplemented with 20 mM ATP and 100 mM DTT for 30 min. at 30°C to allow for phosphorylation. Purified mitochondria were co-incubated with the phosphorylation mixture in import buffer (250 mM sacarose, 10 mM MOPS, 5 mM MgCl_2_, 80 mM KCl and 2.5 mM KPi, pH 7.2) supplemented with 2 mM ATP, 2 mM NADH and 20 mM succinate at 25°C. The reaction was finished by the addition of 25 µM FCCP for 10 min. at 4°C. The samples were centrifuged at 10000×*g* for 20 min. and the pellet and supernatant were prepared for SDS-PAGE.

### Membrane vesicles purification

NIH/3T3 cells were lysed in MSHE buffer as described above. The homogenate was centrifuged for 10 min at 1000×*g*; mitoplasts were isolated from the supernatant and the pellet was resuspended in isolation buffer (250 mM sucrose, 5 mM K-Hepes, 1 mM EGTA, pH 7.4). The suspension was passed four times through a tight fitting pestle and plasma membrane vesicles were purified from a Percoll (GE Healthcare) gradient by ultracentrifugation [38].

### Plasma membrane vesicles and mitoplasts incubation

Plasma membrane fraction and mitoplasts (labelled with MitoTracker Deep Red) were incubated in a 100 µl final volume mix (import buffer). The suspension was co-incubated with 0.3 µg recombinant inactive Akt1 (Prospec), 20 mM ATP and 100 mM MgCl_2_ for 30 min. at 30°C. The mixture was centrifuged at 10000×*g* for 20 min. The pellet was resuspended in 50 mM Tris (plus 0.3% Triton X-100 and 1% BSA).

### Fluorescence labeling

Cells were grown on Lab-Tek Chambered Borosilicate Coverglass System (Nunc) for *in vivo* experiments and transfected with wild type Akt1-GFP or its mutants, Akt1 T308A-GFP or Akt1 S473A-GFP using Lipofectamine 2000 (Invitrogen). Cells were stained with MitoTracker Deep Red (Invitrogen, 100 nM, 45 min at 37°C). At the moment of image acquisition, cells were stimulated with 50 µM H_2_O_2_. For the phosphorylation assay, mitoplasts stained with 5 µM Mitotracker Deep Red (1 h at 37°C) and vesicles were incubated together with 0.3 µg Akt1 in an import assay (30 min at 37°C). The preparations were washed, fixed in 4% paraformaldehyde and resuspended in 50 mM Tris, 0.3% Triton X-100, 1% BSA and co-incubated with primary antiobodies (P-Thr^308^or P-Ser^473^ and ATPase) and secondary antibodies (anti-rabbit Cy2 linked or anti-mouse Cy3 linked). For washing, samples were centrifuged at 10000×*g* for 20 min. Finally, samples were resuspended in PBS and mounted onto coverslides with Fluorsave (Calbiochem).

### Confocal microscopy and image analysis

Images were acquired in an Olympus FV1000 confocal laser scanning microscope with a 60×1.35 NA oil immersion objective. Excitation and filters were as follows: GFP and Cy2, 488 nm excitation, emission BP 500–530 nm; Cy3, 543 nm excitation, emission BP 555–655 nm, MitoTracker Deep Red, 633 nm excitation, emission BP 655–755 nm. Images were acquired in a sequential mode. No channel cross-talk was recovered in any case. The image and statistical analysis was performed with Matlab (MathWorks, Natick, MA) and DIPimage (image processing toolbox for Matlab, Delft University of Technology, The Netherlands). For image analysis, see Methods S5.

### Mass spect

The Akt protein band was excised from a 1D coomassie stained gel and subjected to in-gel tryptic digestion as previously reported [39]. The digest was done in the presence of a mass spectrometry friendly surfactant to provide increased sequence coverage (Protease Max, Promega, Wisconsin) and the reduction steps using DTT was excluded. Samples were alkylated with 10 µM acrylamide (+71 Da) at room temperature for 30 minutes. Extracted peptides were dried to completion and reconstituted to 8 µl in 0.1% formic acid, 2% acetonitrile, 97.9% water. The mass spectrometer was a LCQ Deca XP Plus (Thermo Scientific) which was set in data dependent acquisition mode to perform MS/MS on the top three most intense ions with a dynamic exclusion setting of two (Methods S6).

### Statistical analysis

Data are expressed as means ± SE and analysed by one-way analysis of variance (ANOVA), Dunnett's test and Scheffé test. Statistical significance was accepted at *p* <0.05.

## Supporting Information

Figure S1 High redox status drives cells to apoptosis. Apoptosis was determined by acridine orange and ethidium bromide double staining 48 h after H_2_O_2_ treatment. Morphology and staining were evaluated in a fluorescence microscope (40x).
(1.34 MB TIF)

Figure S2 Loss of mitochondrial membrane potential is involved in H_2_O_2_-triggered apoptosis. The dynamic of the loss of the mitochondrial membrane potential was monitored by the potential-sensitive dye Rho123 under flow cytometry by duplicate in H_2_O_2_ treated and control cells.
(0.38 MB TIF)

Figure S3 Purity controls of the different subcellular fractions by duplicate using specific antibodies against complex I (mitochondria), β-actin (cytosol) and RNA POL II (subunit 250 kDa) (nuclei).
(0.36 MB TIF)

Figure S4 Presence and translocation of hAkt1 and its phosphorylation mutants Akt1 S473A and Akt1 T308A into mitochondria. NIH/3T3 cells transfected with Akt1-GFP, Akt1 S473A-GFP and Akt1 T308A-GFP and stained with MitoTracker Deep Red were stimulated 50 µM H_2_O_2_. Fluorescence intensity of both green (GFP) and red (Mitotracker) channels was followed for 20 min in an Olympus FV1000 confocal microscope. (A) Series of representative merged images after H_2_O_2_ stimulation for Akt and its phosphorylation mutants are shown. An image corresponding to the mitochondrial mask determined by a colocalization algorithm for each image pair is shown on the right. Bar  = 10 µm. (B) Nuclear and cellular masks in which GFP fluorescence change was followed after H_2_O_2_ stimulation (see methods).
(1.99 MB TIF)

Table S1 Mitochondrial membrane potential (Δψmit) was determined by duplicate by measuring Rhodamine 123 fluorescence at 503 nm with a Hitachi F-3010 spectrofluorometer at 37°C. NIH/3T3 mitochondria (0.2 mg/ml) were added to the media and the fluorescence of the suspension was measured. The initial total amount of Rh-123 in the cuvette ([Rh-123]total) and the amount remaining in the media ([Rh-123] out) were used to calculate by subtraction the total amount of Rh-123 taken up by mitochondria ([Rh-123]mit, in nmol/mg protein). Mitochondrial membrane potentials (negative inside) were calculated by the electrochemical Nernst-Guggenheim equation: Δψmit = 59 log ([Rh-123]in/[Rh-123]out). Additions: 8 mM malate (mal); 8 mM glutamate (glu).
(0.03 MB DOC)

Table S2 Complex IV activity was determined by duplicate by recording the oxidation of reducedcytochrome c at 550 nm in the different NIH/3T3 subcellular fractions. Lactate dehydrogenase activity was monitored spectrophotometrically by duplicate in NIH/3T3 subcellular fractions through oxidation of NADH at 340 nm.
(0.03 MB DOC)

Methods S1 (0.02 MB DOC)

Methods S2 (0.03 MB DOC)

Methods S3 (0.03 MB DOC)

Methods S4 (0.03 MB DOC)

Methods S5 (0.03 MB DOC)

Methods S6 (0.02 MB DOC)

Video S1 NIH/3T3 cells transfected with Akt1 T308A-GFP and stained with MitoTracker Deep Red were stimulated 50 µM H_2_O_2_. Fluorescence intensity of both green (GFP) and red (Mitotracker) channels was followed for 20 min in an Olympus FV1000 confocal microscope.
(1.34 MB AVI)

Video S2 Same as Video S1 but redistribution kinetics was followed in a zoomed image as in Fig. 4D.
(0.33 MB AVI)

Video S3 NIH/3T3 cells transfected with Akt1 T308A-GFP and stained with MitoTracker Deep Red were stimulated 50 µM H_2_O_2_. Fluorescence intensity of both green (GFP) and red (Mitotracker) channels was followed for 20 min in an Olympus FV1000 confocal microscope.
(1.16 MB AVI)

Video S4 Same as Video S3 but redistribution kinetics was followed in a zoomed image as in Fig. 4D.
(0.31 MB AVI)
