# Supplementary figures and images for: Akt1 Intramitochondrial Cycling Is a Crucial Step in the Redox Modulation of Cell Cycle Progression
Source: PLoS One. 2009 Oct 21;4(10):e7523. doi: 10.1371/journal.pone.0007523 (PMC2761088; doi:10.1371/journal.pone.0007523)

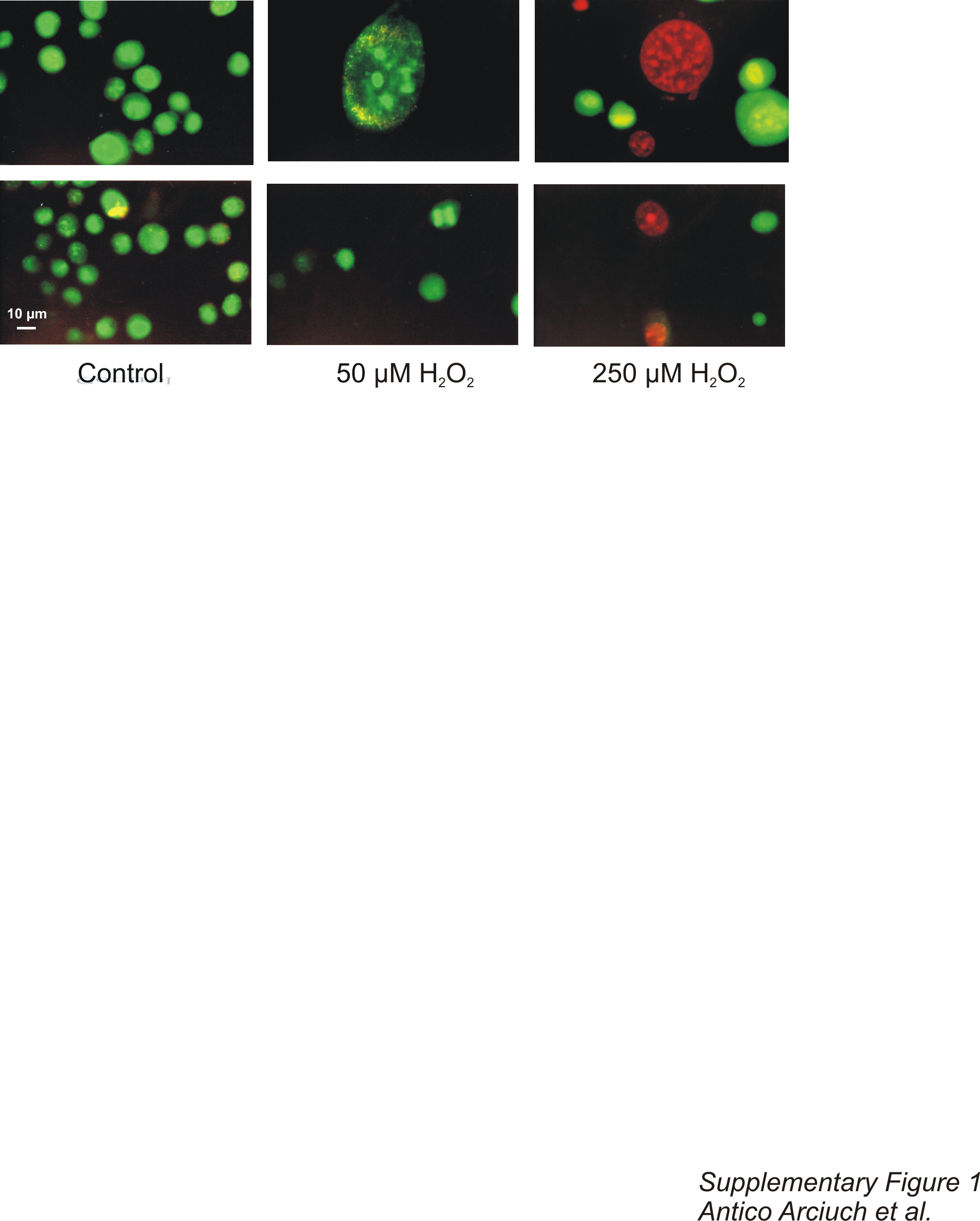

Supplement: Figure S1 — High redox status drives cells to apoptosis. Apoptosis was determined by acridine orange and ethidium bromide double staining 48 h after H2O2 treatment. Morphology and staining were evaluated in a fluorescence microscope (40x). (1.34 MB TIF) [file pone.0007523.s001.tif]

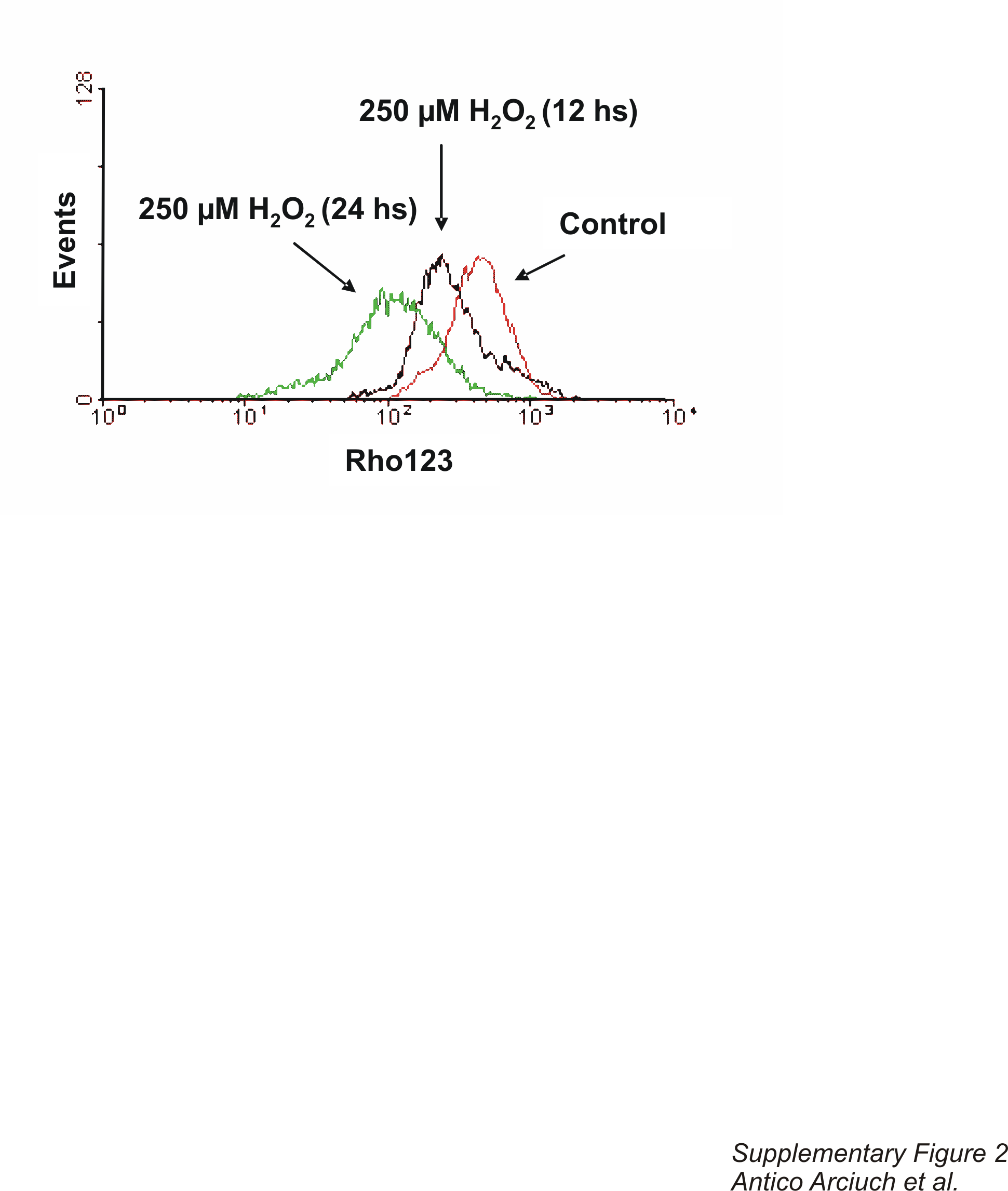

Supplement: Figure S2 — Loss of mitochondrial membrane potential is involved in H2O2-triggered apoptosis. The dynamic of the loss of the mitochondrial membrane potential was monitored by the potential-sensitive dye Rho123 under flow cytometry by duplicate in H2O2 treated and control cells. (0.38 MB TIF) [file pone.0007523.s002.tif]

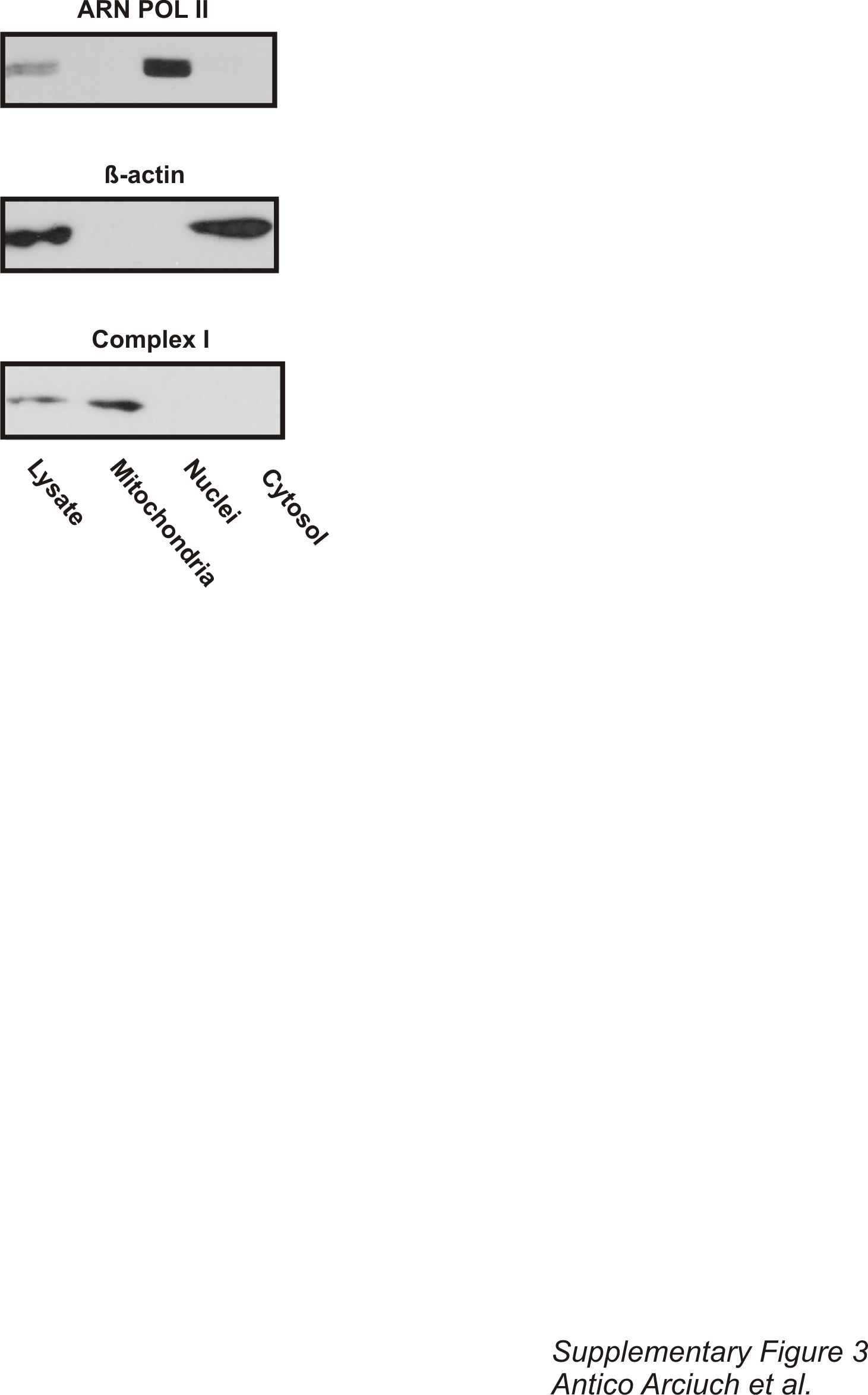

Supplement: Figure S3 — Purity controls of the different subcellular fractions by duplicate using specific antibodies against complex I (mitochondria), β-actin (cytosol) and RNA POL II (subunit 250 kDa) (nuclei). (0.36 MB TIF) [file pone.0007523.s003.tif]

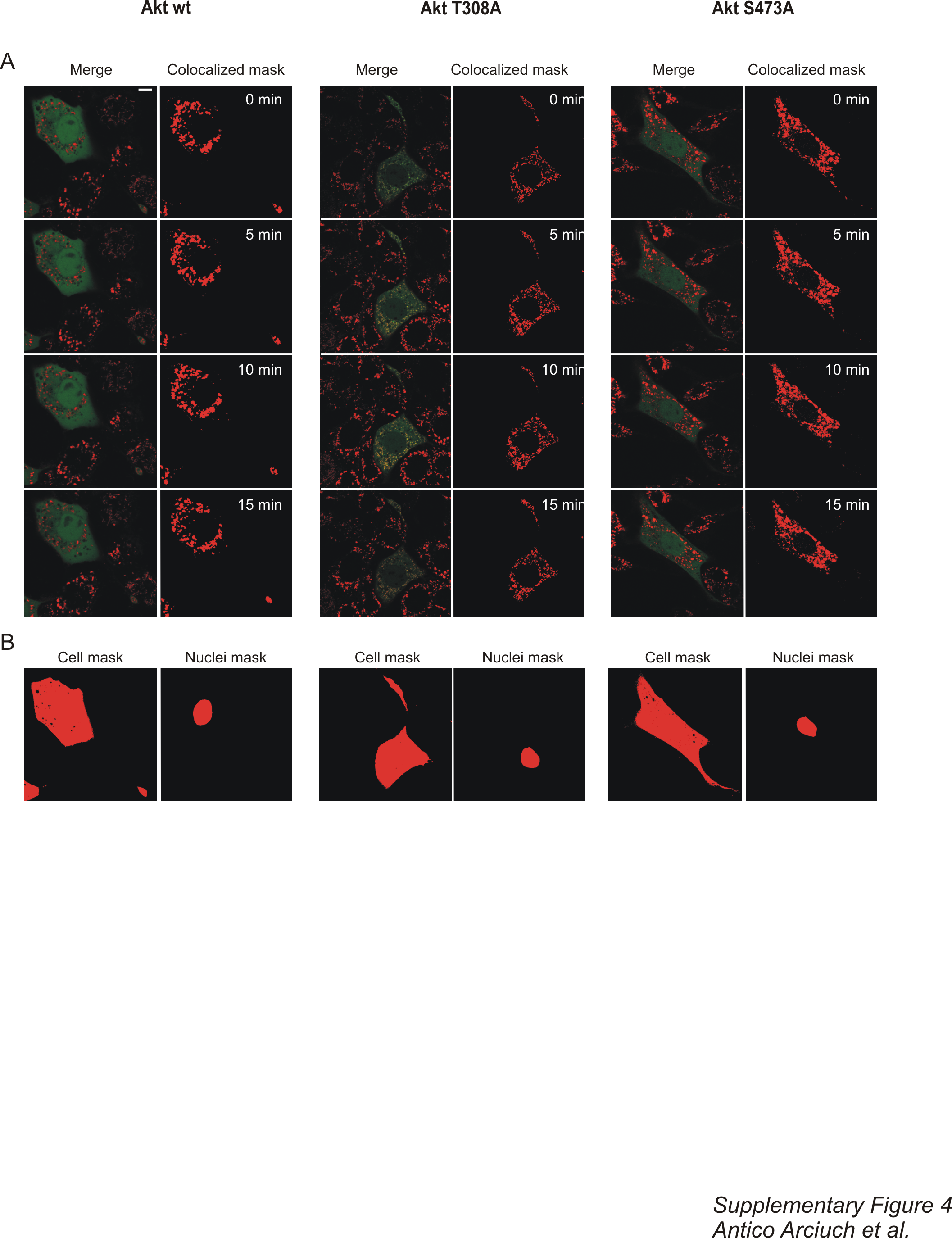

Supplement: Figure S4 — Presence and translocation of hAkt1 and its phosphorylation mutants Akt1 S473A and Akt1 T308A into mitochondria. NIH/3T3 cells transfected with Akt1-GFP, Akt1 S473A-GFP and Akt1 T308A-GFP and stained with MitoTracker Deep Red were stimulated 50 µM H2O2. Fluorescence intensity of both green (GFP) and red (Mitotracker) channels was followed for 20 min in an Olympus FV1000 confocal microscope. (A) Series of representative merged images after H2O2 stimulation for Akt and its phosphorylation mutants are shown. An image corresponding to the mitochondrial mask determined by a colocalization algorithm for each image pair is shown on the right. Bar = 10 µm. (B) Nuclear and cellular masks in which GFP fluorescence change was followed after H2O2 stimulation (see methods). (1.99 MB TIF) [file pone.0007523.s004.tif]
