# Supplementary material for: Akt1 Intramitochondrial Cycling Is a Crucial Step in the Redox Modulation of Cell Cycle Progression
Source: PLoS One. 2009 Oct 21;4(10):e7523. doi: 10.1371/journal.pone.0007523 (PMC2761088; doi:10.1371/journal.pone.0007523)
Supplement: Table S1 — Mitochondrial membrane potential (Δψmit) was determined by duplicate by measuring Rhodamine 123 fluorescence at 503 nm with a Hitachi F-3010 spectrofluorometer at 37°C. NIH/3T3 mitochondria (0.2 mg/ml) were added to the media and the fluorescence of the suspension was measured. The initial total amount of Rh-123 in the cuvette ([Rh-123]total) and the amount remaining in the media ([Rh-123] out) were used to calculate by subtraction the total amount of Rh-123 taken up by mitochondria ([Rh-123]mit, in nmol/mg protein). Mitochondrial membrane potentials (negative inside) were calculated by the electrochemical Nernst-Guggenheim equation: Δψmit = 59 log ([Rh-123]in/[Rh-123]out). Additions: 8 mM malate (mal); 8 mM glutamate (glu). (0.03 MB DOC) [file pone.0007523.s005.doc]

**Table S1. Membrane potential of NIH/3T3 mitochondria in state 4 on the basal condition and at high redox status (250 µM H_2_O_2_).**

| **Experimental condition** | **Membrane potential (mV)** |
| --- | --- |
| Control cell mitochondria  + mal-glu (state 4) | -185.58 |
| 250 µM H_2_O_2_ cell mitochondria  + mal-glu (state 4) | -151.07 |

Mitochondrial membrane potential (Δψ_mit_) was determined by duplicate by measuring Rhodamine 123 fluorescence at 503 nm→527 nm with a Hitachi F-3010 spectrofluorometer at 37°C. NIH/3T3 mitochondria (0.2 mg/ml) were added to the media and the fluorescence of the suspension was measured. The initial total amount of Rh-123 in the cuvette ([Rh-123]_total_) and the amount remaining in the media ([Rh-123] _out_) were used to calculate by subtraction the total amount of Rh-123 taken up by mitochondria ([Rh-123]_mit_, in nmol/mg protein). Mitochondrial membrane potentials (negative inside) were calculated by the electrochemical Nernst–Guggenheim equation: Δψ=59 log ([Rh-123]_in_/ [Rh-123]_out_). Additions: 8 mM malate (mal); 8 mM glutamate (glu).
