# Supplementary material for: Akt1 Intramitochondrial Cycling Is a Crucial Step in the Redox Modulation of Cell Cycle Progression
Source: PLoS One. 2009 Oct 21;4(10):e7523. doi: 10.1371/journal.pone.0007523 (PMC2761088; doi:10.1371/journal.pone.0007523)
Supplement: Table S2 — Complex IV activity was determined by duplicate by recording the oxidation of reducedcytochrome c at 550 nm in the different NIH/3T3 subcellular fractions. Lactate dehydrogenase activity was monitored spectrophotometrically by duplicate in NIH/3T3 subcellular fractions through oxidation of NADH at 340 nm. (0.03 MB DOC) [file pone.0007523.s006.doc]

|  | **Mitochondria** | **Cytosol** | **Nuclei** |
| --- | --- | --- | --- |
| **Complex IV** (*k*´/min mg prot) | 19.2 | 1.8 | 1.5 |
| **Lactate dehydrogenase**  (nmol NADH/mg prot) | 24 | 156 | 18 |

**Table S2. Enzimatic activities of the different subcellular fractions.**

Complex IV activity was determined by duplicate by recording the oxidation of reduced cytochrome *c* at 550 nm in the different NIH/3T3 subcellular fractions. Lactate dehydrogenase activity was monitored spectrophotometrically by duplicate in NIH/3T3 subcellular fractions through oxidation of NADH at 340 nm.
