# Supplementary material for: Akt1 Intramitochondrial Cycling Is a Crucial Step in the Redox Modulation of Cell Cycle Progression
Source: PLoS One. 2009 Oct 21;4(10):e7523. doi: 10.1371/journal.pone.0007523 (PMC2761088; doi:10.1371/journal.pone.0007523)
Supplement: Methods S1 — (0.02 MB DOC) [file pone.0007523.s007.doc]

**Apoptosis assay**

Apoptosis was additionally investigated through acridine orange (100 µg/ml)/ethidium bromide (100 µg/ml) staining by fluorescence microscopy (40X).
