# Supplementary material for: Akt1 Intramitochondrial Cycling Is a Crucial Step in the Redox Modulation of Cell Cycle Progression
Source: PLoS One. 2009 Oct 21;4(10):e7523. doi: 10.1371/journal.pone.0007523 (PMC2761088; doi:10.1371/journal.pone.0007523)
Supplement: Methods S2 — (0.03 MB DOC) [file pone.0007523.s008.doc]

**Spectrofluorometric determination of mitochondrial membrane potential**

Mitochondrial membrane potential (Δψ_mit_) was determined by measuring Rhodamine 123 (Rho123) fluorescence at 503 nm→527 nm with a Hitachi F-3010 spectrofluorometer at 37°C. The fluorescence of the media (150 mM sucrose, 4 mM MgCl_2_, 5 mM potassium phosphate, 30 mM KOH-HEPES pH 7.4) containing 0.1 μM Rho123 was determined before addition of mitochondria. This measurement was used as an indication of the total dye concentration ([Rho123]_total_, in nmol/μl). NIH/3T3 mitochondria (0.2 mg/ml) were added to the media in the presence of 8 mM malate and 8 mM glutamate. After an equilibrium had been reached, the fluorescence of the suspension was measured and the contents of the cuvette were centrifuged at 15000×*g* to pellet the mitochondria. The Rho123 concentration remaining in the media ([Rho123]_out_, in nmol/μl) was calculated from the fluorescence values of the supernatant. The initial total amount of Rho123 in the cuvette ([Rho123]_total_) and the amount remaining in the media ([Rho123]_out_) were used to calculate by subtraction the total amount of Rho123 taken up by mitochondria ([Rho123]_mit_, in nmol/mg protein). The concentration of free Rh-123 in the matrix ([Rho123]_in_, in nmol/μl) was calculated using the following equation, and the binding partition coefficients at 37°C (K_i_=26 μl/mg, K_o_=120 μl/mg): [Rho123]_mit_ = K_i_ [Rho123]_in_ + K_o_ [Rho123]_out_ Mitochondrial membrane potentials (negative inside) were calculated by the electrochemical Nernst-Guggenheim equation: Δψ = 59 log ([Rho123]_in_/[Rho123]_out_).
