# Supplementary material for: Akt1 Intramitochondrial Cycling Is a Crucial Step in the Redox Modulation of Cell Cycle Progression
Source: PLoS One. 2009 Oct 21;4(10):e7523. doi: 10.1371/journal.pone.0007523 (PMC2761088; doi:10.1371/journal.pone.0007523)
Supplement: Methods S3 — (0.03 MB DOC) [file pone.0007523.s009.doc]

**Measurement of mitochondrial membrane potential**

The Δψ_mit_ was also measured by flow cytometry. Cells were grown at a density of 5 × 10^4^ cells in 6-well plates. After incubation with 250 µM H_2_O_2_ for 12 and 24 h, treated and control cells were collected and washed with PBS three times. Cells in 1 ml PBS were stained with 1 μM Rho123 for 15 min in dark at 37°C. Cells were collected by centrifugation, washed with PBS and then resuspended in 0.5 ml PBS. Fluorescence emitted from the Rho123 was detected with a FACScalibur flow cytometer (Becton-Dickinson, Mountain View, CA) and analyzed with WinMDI software for Windows.
