# Supplementary material for: Akt1 Intramitochondrial Cycling Is a Crucial Step in the Redox Modulation of Cell Cycle Progression
Source: PLoS One. 2009 Oct 21;4(10):e7523. doi: 10.1371/journal.pone.0007523 (PMC2761088; doi:10.1371/journal.pone.0007523)
Supplement: Methods S4 — (0.03 MB DOC) [file pone.0007523.s010.doc]

**Enzymatic marker activities**

Complex IV (cytochrome oxidase) activity was determined by recording the oxidation of 50 µM reduced cytochrome *c* at 550 nm (ε_550_ = 21 mM^-1^cm^-1^) at 30^o^C using 0.1 mg protein/ml in a Hitachi U3000 spectrophotometer. The rate of the reaction was determined as the pseudo-first-order reaction constant, k´, and expressed per min per mg protein. Lactate dehydrogenase activity was assayed spectrophotometrically by following NADH oxidation at 340 nm. Twenty-five micrograms of protein were added to 100 mM phosphate buffer, pH 7.0 in the presence of 1 mM piruvic acid, 0.1% triton X-100 and 0.22 mM NADH; ε_340_ = 6.22 mM^-1^cm^-1^.
