# Supplementary material for: Akt1 Intramitochondrial Cycling Is a Crucial Step in the Redox Modulation of Cell Cycle Progression
Source: PLoS One. 2009 Oct 21;4(10):e7523. doi: 10.1371/journal.pone.0007523 (PMC2761088; doi:10.1371/journal.pone.0007523)
Supplement: Methods S5 — (0.03 MB DOC) [file pone.0007523.s011.doc]

**Supporting methods**

**Image analysis**

To evaluate Akt1-GFP and its mutants redistribution among the subcellular compartments, a mitochondrial mask was performed using MitoTracker fluorescence intensity or an algorithm to detect and quantify colocalization (Villalta *et al.*, in preparation), nuclear masks were done manually, and cellular masks were obtained over transfected cells using GFP fluorescence intensity. Akt1-GFP fluorescence intensity was quantified in each compartment and normalized by the mean fluorescence intensity of the whole cell or group of cells simultaneously imaged. Manders overlap map and Pearson’s correlation coefficient map were determined by estimating each single pixel pair contribution, divided by the complete denominator of the coefficient equation. For imaging in time, cells were stimulated with H_2_O_2_ and imaged every 1-2 min over a period of 20-30 min at room temperature; 3 to 7 equidistant (0.5 μm) planes were evaluated for each cell or group of cells (an image set corresponds to the images over time of all the planes, in both the green and red channels, for a cell or group of cells simultaneously imaged). To follow the redistribution of Akt and its mutant variants in the cell, we delimited three subcellular compartments: mitochondria, cytosol and nuclei. Most of the pixels in the images were background and displayed low fluorescence intensity. Thus, the mean fluorescence intensity of the whole image corresponded mainly to the intensity of background. The cellular compartment was delimited when GFP fluorescence intensity was over twice the mean fluorescence intensity of the whole GFP image (Fig. S4). Nuclear masks were performed manually (Fig. S4). To define the mitochondrial compartment we selected a mask determined by a MitoTracker fluorescence intensity twice above the mean of the whole image and combined it with the cellular mask in order to evaluate only transfected cells (Galli *et al.*, submitted). Otherwise, we used a novel algorithm to detect and quantify colocalization in a pair of fluorescence confocal images. The algorithm sequentially detects the colocalizing and anti-colocalizing populations of pixels and returns a mask which encompasses those pixels that significantly display fluorescence intensity in both channels (Villalta *et al.*, in preparation). In our case, these masks corresponded to pixels which displayed both Akt-GFP and MitoTracker Deep Red fluorescence (Fig. S4). Cytosolic compartment was determined by the remaining cell area after subtracting mitochondrial and nuclear masks to the whole cell mask. The change in GFP fluorescence intensity was evaluated in every compartment in time (Akt1 T308A-GFP 4 image-sets, 3 independent experiments; Akt1 S437A-GFP 5 image-sets, 3 independent experiments; wild type Akt-GFP, 4 image-sets, 3 independent experiments) and normalized by the mean fluorescence intensity of the whole cell or group of cells simultaneously imaged. Colocalization coefficients and maps:

**Manders overlap coefficient (*R*) and Pearson´s correlation coefficient (*r*) are** defined by

$R=\frac{\sum S1i\times S2i}{\sqrt{\sum(S1i)^{2}\times\sum(S2i)^{2}}}$; *Eq1*

$r=\frac{\sum(S1i-S1mean)\times(S2i-S2mean)}{\sqrt{\sum(S1i-S1mean)^{2}\times(S2i-S2mean)^{2}}}$; *Eq 2*

where S1*i* represents signal intensity of pixels in the channel green and S2*i* represents signal intensity of pixels in the channel red; S1*mean* and S2*mean* reflect the average intensities of these respective channels. *R* and *r* maps are constructed as follows: every single pixel pair value corresponds to the value they generate according to the equation 1 or 2, respectively, divided the whole denominator of the equation. Thus, if we consider an image an array of *n*×*m* pixels, then the maps are generated as follows:

***R map***

$$\underline{\left[\begin{matrix} \frac{S11,1\times S21,1}{\sqrt{\sum(S1i)^{2}\times\sum(S2i)^{2}}};..........\frac{S11,n\times S22,n}{\sqrt{\sum(S1i)^{2}\times\sum(S2i)^{2}}} \\ . \\ . \\ . \\ \frac{S1m,1\times S2m,1}{\sqrt{\sum(S1i)^{2}\times\sum(S2i)^{2}}};..........\frac{S1m,n\times S2m,n}{\sqrt{\sum(S1i)^{2}\times\sum(S2i)^{2}}} \end{matrix}\right]}$$

***r map***

For evaluation of Akt Ser or Thr phosphorylation in vesicle and mitoplast preparations we performed a mask over Cy3 (vesicle marker) fluorescence intensity channel or over Mitotracker Deep Red (mitoplast marker) fluorescence intensity channel (mask was achieved when the fluorescence intensity of each channel was above 50 times the mean intensity of the respective channel) and combined both masks. The final mask contained pixel groups (particles) that displayed fluorescence in the Cy3 channel (which acquainted for vesicles), in the MitoTracker channel (which acquainted for mitoplasts) or in both channels (which acquainted for mitoplasts engulfed by vesicles). Cy2 (P-Ser or P-Thr marker), Cy3 (ATPase marker) and Mitotracker fluorescence intensities were evaluated in these masks for every single particle. A total of 20 images were analyzed for P-Ser quantification and 15 images for P-Thr quantification (~900 particles for each case). Thus, we obtained a final data matrix of three fluorescence intensity values per particle, for each Akt phosphorylation site. These data matrices were analized by *k*-means clustering, a method of [cluster analysis](http://en.wikipedia.org/wiki/Cluster_analysis) which aims to [partition](http://en.wikipedia.org/wiki/Partition_of_a_set) *n* observations into *k* clusters in which each observation belongs to the cluster with the nearest [mean](http://en.wikipedia.org/wiki/Mean). The *k*-means clustering algorithm is integrated as a Matlab function (MathWorks, Natick, MA). We separated the dots into 6 populations. From these populations we selected three representative groups in accord to their predominant label: mainly vesicles (mainly ATPase-Cy3 staining), mainly mitoplasts (mainly MitoTracker Deep Red staining) or both vesicles and mitoplasts together (both Cy3 and MitoTracker fluorescence intensities), and compared either P-Akt1 Ser^473^-Cy2 or P- Akt1 Thr^308^-Cy2 fluorescence among these populations. There was always a mitotracker stained population with no stain for Cy2: presumably no Akt import occurred in these particles.
