# Supplementary material for: Akt1 Intramitochondrial Cycling Is a Crucial Step in the Redox Modulation of Cell Cycle Progression
Source: PLoS One. 2009 Oct 21;4(10):e7523. doi: 10.1371/journal.pone.0007523 (PMC2761088; doi:10.1371/journal.pone.0007523)
Supplement: Methods S6 — (0.02 MB DOC) [file pone.0007523.s012.doc]

**Mass spect**

Nano reversed phase HPLC was done at the University of Standford CA, U.S.A by using an Eksigent 2D nanoLC (Eksigent, Dublin, CA) with buffer A consisting of 0.1 % formic acid in water and buffer B 0.1 % formic acid in acetonitrile. A fused silica column self packed with duragel C18 (Peeke, Redwood City, CA) matrix was used with a linear gradient from 5 % B to 40 % B over 60 minutes at a flow rate of 450 nL/minute. The nano-HPLC was interfaced with an Advion Nanomate (Ithaca, NY) for nanoESI into the mass spectrometer. The mass spectrometer was a LCQ Deca XP Plus (Thermo Scientific) which was set in data dependent acquisition mode to perform MS/MS on the top three most intense ions with a dynamic exclusion setting of two. The DTA files were extracted from the raw data using the bioworks browser (Thermo Scientific). The data was searched against the Human NCBI database using Mascot allowing for variable modifications of propionamide (C), oxidation (M), sufenic acid (C) and cysteic acid (C).
